# Modern Developments for Textile-Based Supercapacitors

**DOI:** 10.1021/acsomega.3c01176

**Published:** 2023-03-30

**Authors:** Samantha Newby, Wajira Mirihanage, Anura Fernando

**Affiliations:** Department of Materials, Faculty of Science and Engineering, University of Manchester, Engineering Building A, Manchester M13 9PL, U.K.

## Abstract

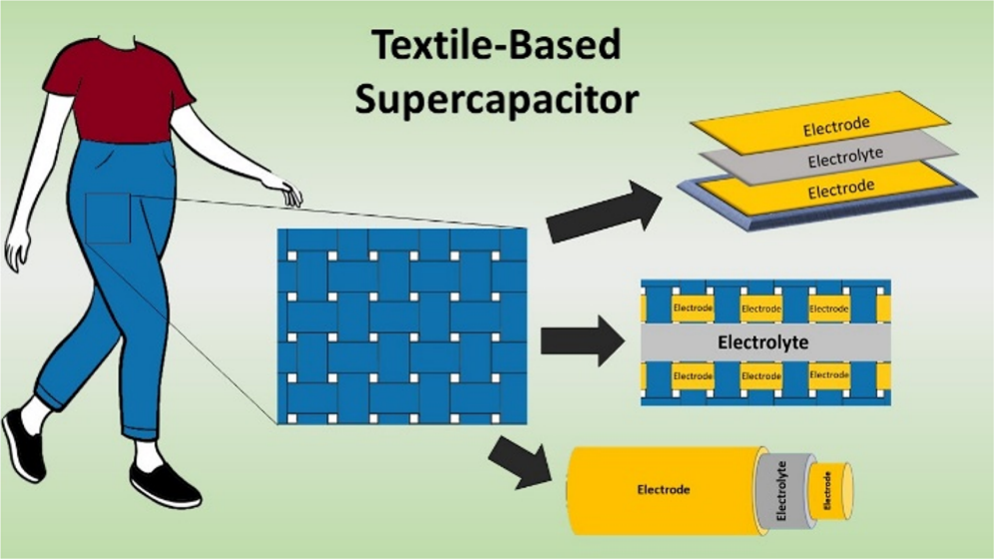

Smart textiles are transforming the future of wearable technology, and due to that, there has been a great deal of new research looking for alternative energy storage. Supercapacitors offer high discharge rates, flexibility, and long life cycles and can be integrated fully into a textile. Optimization of these new systems includes utilizing electrically conductive materials, employing successful electrostatic charge and/or faradaic responses, and fabricating a textile-based energy storage system without disrupting comfort, washability, and life cycle. This paper examines recent developments in fabrication methods and materials used to create textile supercapacitors and what challenges still remain.

## 1 Introduction

Smart textiles are increasingly popular due to their abilities to sense, react, and adapt to stimuli in the surrounding environment while being comfortable and relatively unseen when fabricated into clothing. Research into flexible, effective energy storage systems has become crucial to the life cycle and continued wearability of these devices. Currently, textile batteries and supercapacitors have been researched.^1−6^ These systems not only need to hold and deliver energy but also need to maintain certain properties of a textile such as deformation and stretchability. While initial research into wearable energy storage systems focused on applying rigid storage systems to a textile surface, resulting in stiff and unyielding textiles, new research looks at seamlessly integrating the energy system into the textile. This can be done by adding a flexible system to the textile or incorporating the system into the textile itself.

Batteries have been made into lightweight, flexible, and comfortable devices, but in order to work in textiles, the battery has to be nontoxic. This limits the conventional methods for construction and requires new materials to be researched. Printed batteries allow for thin, flexible devices that can easily be fabricated onto a textile.^7^ Printing methods for both alkaline and lithium batteries include inkjet printing,^8,9^ dispenser printing,^10,11^ and screen printing.^12,13^ Alternative methods of fabricating textile-based batteries include spinning the battery and encapsulating it in the electrolyte material^14^ or coating a textile with an electrolyte and sandwiching that between the cathode and anode material.^15^ Lithium-ion batteries have also been fabricated into liquid-activated batteries that are useful for textiles such as life jackets.^16^ Researchers have also manufactured a fiber-shaped lithium-ion battery that can be knitted or woven into a textile for mass production of a wearable battery.^17^ However, textile batteries tend to have lower power densities and life cycle than supercapacitors.

Supercapacitors are being researched for wearable storage devices as they offer higher power densities, structural flexibility, and specific capacitance and can be seamlessly integrated into the textile.^18−22^ Supercapacitors have the potential to be added to technical textiles to power sensors for medical, sport fitness, and other monitoring devices. Their high power densities mean that the textile supercapacitors can be small and still manage to power the required devices in the textile, while batteries may require more space in the textile to create the same amount of power. The long life cycle also allows the textile devices to last longer without replacing the energy storage unit. However, supercapacitors are restricted by low energy densities and short energy-holding capabilities.^23,24^ These drawbacks are being overcome with the use of new materials, better fabrication methods, and more efficient structural designs. This paper reviews recent textile supercapacitor systems and how their design and integration can influence the capacitance, energy density, and power density of the system.

## 2 Textile Supercapacitors

**Figure 1:**
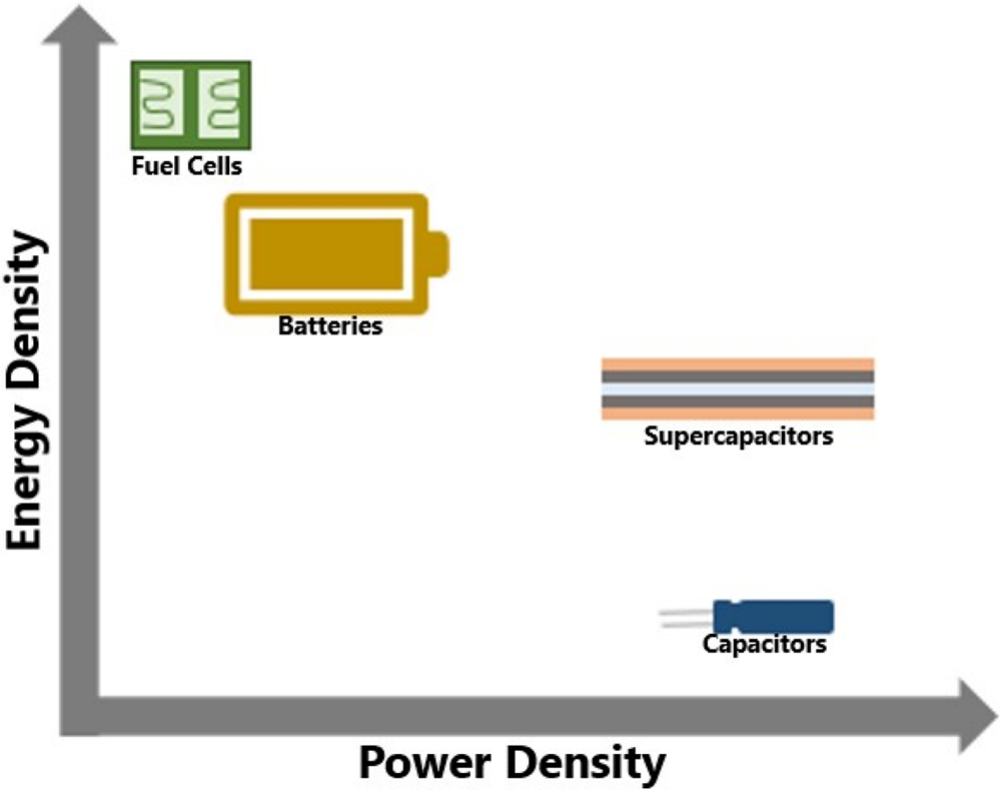
Energy power sources with relation to energy and power densities.

Textile supercapacitors not only need to exhibit high power, long life cycle, stability, and good energy density but also have to deform, stretch, be biocompatible, and be washable in order to work as a wearable device. While supercapacitors exhibit high power densities, they currently do not match batteries on the energy density, as seen in Figure 1.^25^ Also, the textile structures used in these devices may offer inherent flexibility and some stretch, but if the conductive material is not chosen and fabricated correctly, the device will be brittle, degrade during use, and not uphold the electrochemical performance necessary to power a sensor or actuator. Textile supercapacitors have notably low mechanical strength and durability compared to other supercapacitors, and they are currently very expensive to fabricate.^26,27^ Therefore, the design of the supercapacitor, the fabrication methods used, and the materials chosen all need to be considered to increase the textile and electrochemical performance of the supercapacitor.

### 2.1 Characteristics

**Figure 2:**
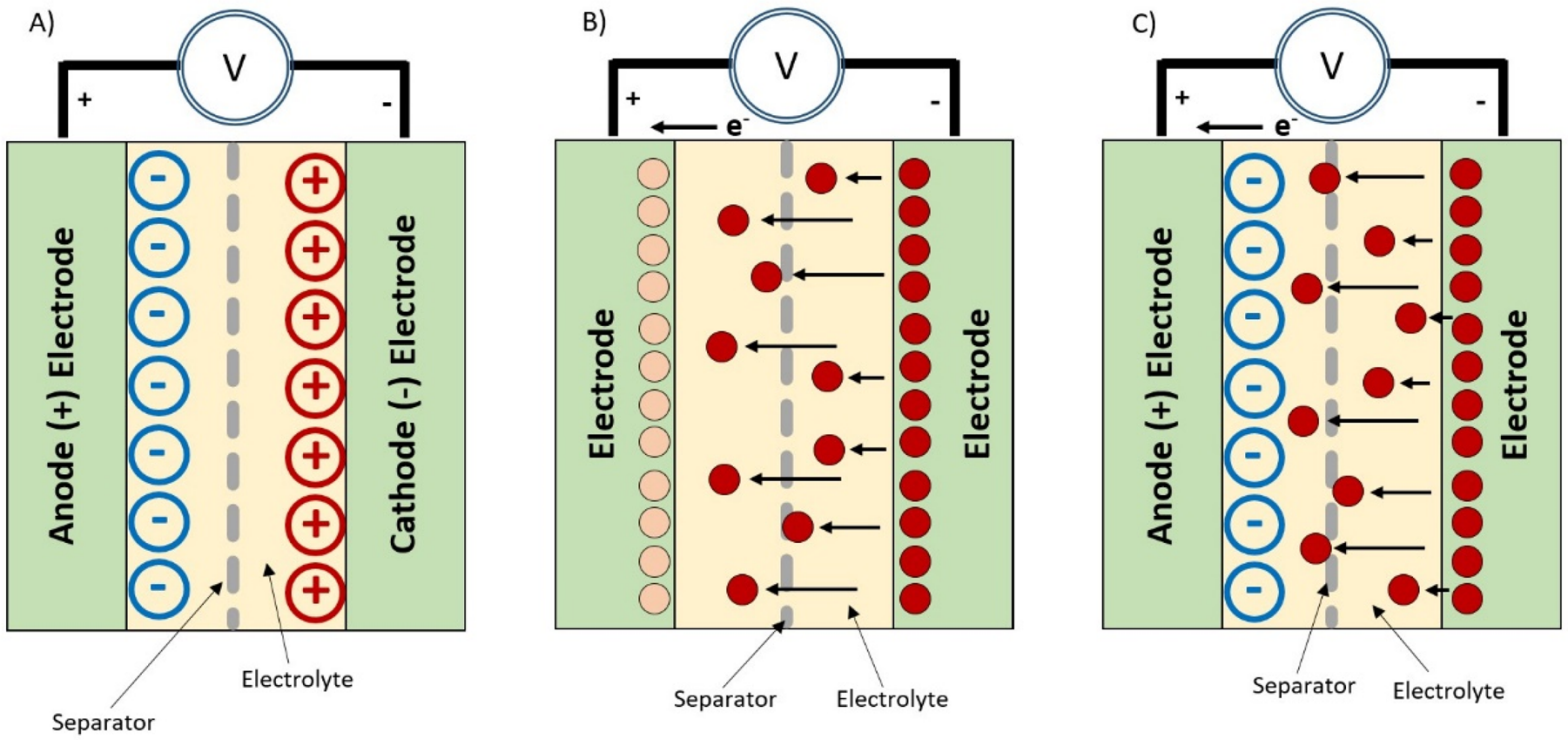
Supercapacitor designs. (a) EDLC, (b) pseudocapacitor, and (c) hybrid.

Supercapacitors come in three types based on their storage principles: electrical double-layer capacitors (EDLCs), pseudocapacitors, and hybrid supercapacitors, as shown in Figure 2. EDLCs are similar to lithium ion batteries and store electrical energy through static charges amassed from the adsorption of both the anions and cations within the electrodes.^28,29^ EDLCs are usually constructed from carbon-based materials, such as graphene or carbon nanotubes, because the high surface area given from the porous material can create large active sites for electrons and, therefore, high-density capacitors.^30^ Pseudocapacitors rely on faradaic and electrochemical responses between the electrolyte and the active electrode material to store energy and are made from conductive polymers or metal oxides.^31^ Hybrid supercapacitors combine the two and are the preferred type for textile supercapacitors.^5,21,32−34^

Because of the limited amount of material that becomes a textile supercapacitor, the amount of surface area and potential for faradaic responses are key factors for determining capacitance.^35^ The specific capacitance of a supercapacitor’s discharge is the electrochemical performance and capability of the device, which can be determined from the equation
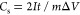
1In a textile supercapacitor, the mass is usually the only active material, either a single electrode or the entire device.^36^ The energy density is the energy stored per unit volume and can be calculated by the equation^37^
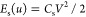
2Using this, the power density of that single fiber can be calculated by the equation^37^
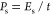
3

Finally, the equation that will determine how the supercapacitor performs is the maximum power. This focuses on the internal makeup of the supercapacitor and is represented by the equation^38^
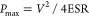
4

A higher resistance equals a lower efficiency in power, so a low ESR, and therefore a large voltage window, is desired. Low resistance can be achieved by highly conductive materials and junctions in the device that do not fracture or reduce the conductivity within the device.

Textile supercapacitors can be made with symmetric, asymmetric, or hybrid electrodes. Symmetric supercapacitors utilize two similar electrodes that can offer both EDLC and faradaic properties.^32,39,40^ Asymmetric supercapacitors feature two electrodes with differing material.^41−43^ Hybrid systems require an anionic and a cationic electrode. For the anionic and cationic electrodes, there has to be a minimum resistance to achieve maximum capacitance. Therefore, the formula
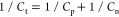
5can be used.^38^ Finally, the electrolyte, which affects the device’s capacitance, the energy and power densities, and stability, is found between the two electrodes and should have a high voltage window, high ionic concentration, low viscosity, and low resistivity.^28^

Outside of the characteristics and properties that make a supercapacitor function, the textile supercapacitor must also maintain textile characteristics. The textile must offer deformation, stretchability, washability, and wearability while being nontoxic to humans and maintaining conductivity. To test these characteristics and make sure the textile supercapacitor can last over time, certain tests can be done. Tensile testing can determine the breaking point of the device or how its properties endure over time when bent or under strain. If the device is not stretchable, this device will fail when added to textiles that are in motion. Washability tests can look at how well the device withstands water and washing. If the device dissolves, cracks, or otherwise is damaged by water or drying, it will not work for textile application.

## 3 Fabrication

**Figure 3:**
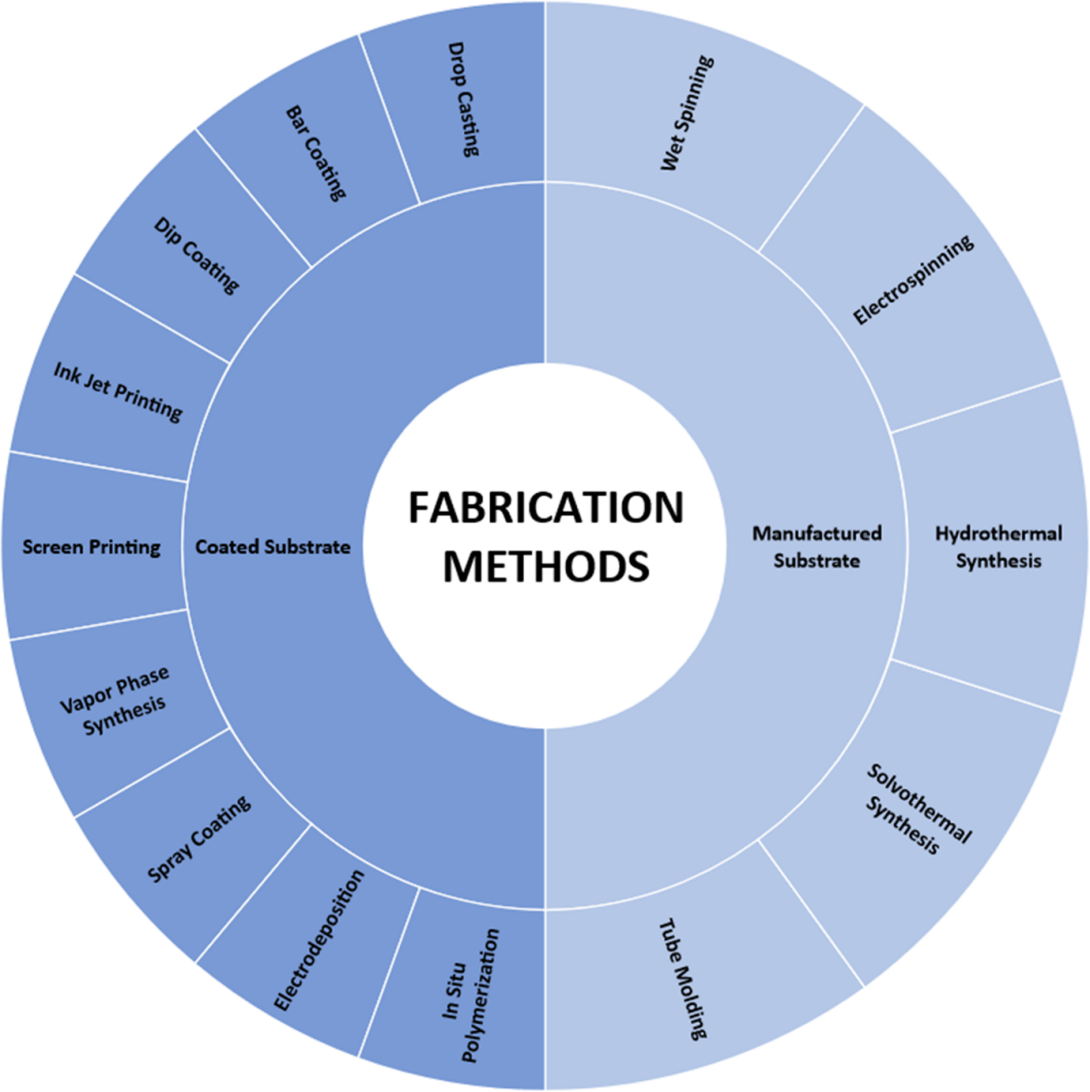
Fabrication methods for textile-based supercapacitors.

There are several methods to fabricate a textile supercapacitor. Depending on the stage of integration, such as textile/yarn or fiber level, several different methods can be used, as seen in Figure 3. Researchers have found that both coating a substrate in conductive material and manufacturing a new fiber or monofilament work for creating textile supercapacitors. Different textile substrates offer properties that can assist in fabricating the supercapacitor. Natural fibers, such as cotton, offer porous structures that, when coated, give large amounts of surface area for the electrode material or can offer a porous electrolyte structure that allows materials to be injected into the structure to assist in capacitance. Textiles also offer intrinsic flexibility, stretch, and strength in their structures from being knitted or woven which helps supercapacitor systems become wearable. Conductive material can also be spun into fibers or filaments and designed to offer porosity. Table 1 shows some fabrication methods alongside their materials and resulting properties.

**Table 1:** Supercapacitor Fabrication Methods and Properties

Fabrication Method	Electrode	Electrolyte	Specific Capacitance	Energy Density	Power Density	Retention
Drop Casting^44^	PEDOT:PSS	Sweat	7.64 F/g	1.36 Wh/kg	329.70 W/kg	75% after 4000 cycles
Bar Coating^45^	Cloth, graphene, CTAB, and NiO:Yb	PMMA/H_3_PO_4_	8.00 F/g	230.60 Wh/kg	50.60 W/kg	45% after 500 cycles
Inkjet Printing^46^	PP/Au/PPy	H_2_SO_4_/PVA	72.30 F/g	6.12 Wh/kg	139.00 W/kg	55.4% after 2000 cycles
Screen Printing^47^	Ag/PPy/MnO^2^ and activated carbon	PVA-Na_2_SO_4_	95.30 mF/cm^2^	0.0337 mWh/cm^2^	0.38 mW/cm^2^	90.8% after 5000 cycles
Vapor Phase Synthesis^48^	PEDOT/graphite and graphite/PU	H_2_SO_4_	798.20 mF/cm^2^	27.70 μWh/cm^2^	0.051 mW/cm^2^	101% after 10000 cycles
Wet Spinning^49^	Ti_3_C_2_T_*x*_/RGO-0.9	H_2_SO_4_	415.00 F/cm^3^	3.40 mWh/cm^3^	10.20 mW/cm^3^	100% after 500 cycles
Electrospinning^50^	PAN-PMAA-Co_3_O_4_	Na_2_SO_4_	125.00 F/g	8.90 W/kg	725.00 W/kg	90% after 7000 cycles
In Situ Polymerization^51^	r-PANI-GOF	H_2_SO_4_/PVA	At max 1755.00 mF/cm^2^	42.76 μW/cm^2^	40.19 μW/cm^2^	73% after 500 cycles
Tube Molds^52^	PEDOT:PSS/PANI, Ag/AgCl, and Pt	H_2_SO_4_	367.70 F/g	42.40 Wh/kg	302.30 W/kg	71.6% after 2000 cycles
Solvothermal Coprecipitation^31^	PEDOT–PSS/NMCO	KOH	1234.50 F/g	51.90 Wh/kg	275.00 W/kg	83.7% after 1000 cycles

**Figure 4:**
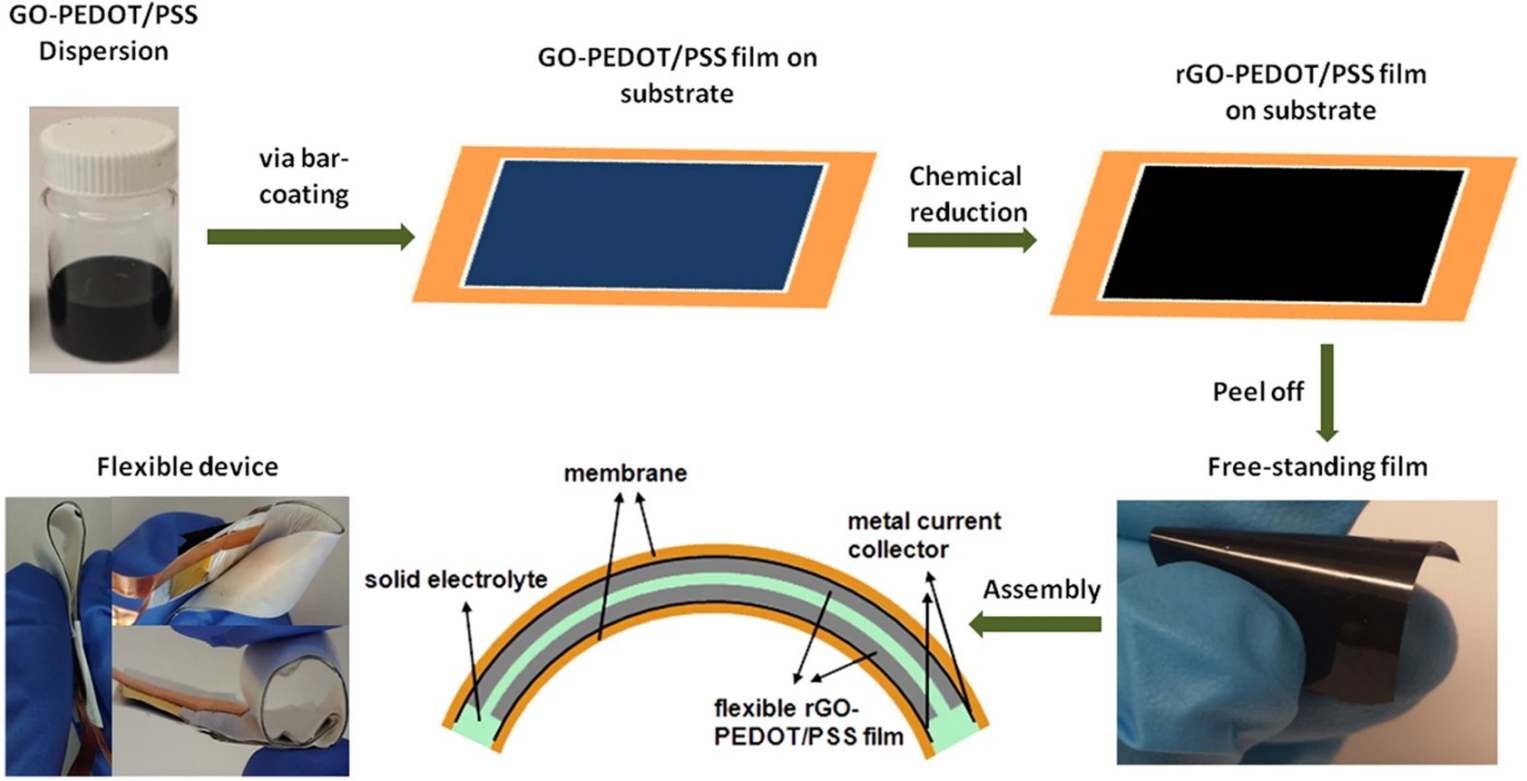
Graphical representation of bar-coating supercapacitors with an image of the final product. Reprinted with permission from Liu, Y.; Weng, B.; Razal, J. M.; Xu, Q.; Zhao, C.; Hou, Y.; Seyedin, S.; Jalili, R.; Wallace, G. G.; Chen, J., High-Performance Flexible All-Solid-State Supercapacitor from Large Free-Standing Graphene-PEDOT/PSS Films. *Sci. Rep.***2015**, *5* (1), 17045. Copyright 2015, Scientific Reports (CC by 4.0).

Considered some of the easiest coating methods, drop casting, bar coating, dip coating, and painting are facile and can be easily scalable. Drop casting is when a conductive ink is dropped onto the substrate, and as the solution evaporates, a conductive film forms on the substrate.^44,53−55^ It can be adapted for various materials and substrates and does not require specific equipment. However, it is difficult to control the thickness of the material and does not lend itself to uniformity. While it can result in high cycling stability,^54^ it is not an easily scalable method. Therefore, to control the ink deposition, bar coating can be used. Bar coating deposits the ink onto a substrate and uses a bar to remove the excess solution to make a uniform coating.^45,56^ This can result in thin film substrates with large amounts of specific surface area that increases the active site for electrons, as shown by Figure 4. One research group used a cotton fabric, featuring high porosity and potential surface area, and deposited graphene and ytterbium/nickel (YbNi) onto it to boost the electrochemical performance of their device to achieve a supercapacitor that lasts more than 30 min longer during discharging than devices without the YbNi.^45^

Dip coating is a method that involves fully saturating a textile or fiber/filament in a conductive solution.^21,57−63^ This method is easy to use, cost effective, and highly scalable and potentially gives the device bendability and stretchability depending on what is coated. Researchers can use a dip-pad-dry method, which involves dipping the substrate in the solution, removing the excess solution, and drying the substrate. Dip coating can coat an entire fabric, similarly to dyeing the fabric or dipping the yarn or filament in the solution to the knit or be woven. A group led by Mao created a triboelectric system partnered with a supercapacitor made from dipped yarn that was then encapsulated with polydimethylsiloxane (PDMS) to prevent degradation through wear and washing.^59^ The encapsulation, notably, did not affect the bendability of the system, and even after washing, the system was bendable and conductive. If a textile is dip coated, the fabric can then be used to form the electrode material. The electrolyte can then be added on top of the fabric, or the fabric can be placed on either side of the electrolyte in a sandwich structure. This has been done to create a belt-like supercapacitor with an energy density of 0.2 μWh/cm^2^, a power density of 0.09 mW/cm^2^, and a retention rate of 93.6% after 5000 cycles.^21^

**Figure 5:**
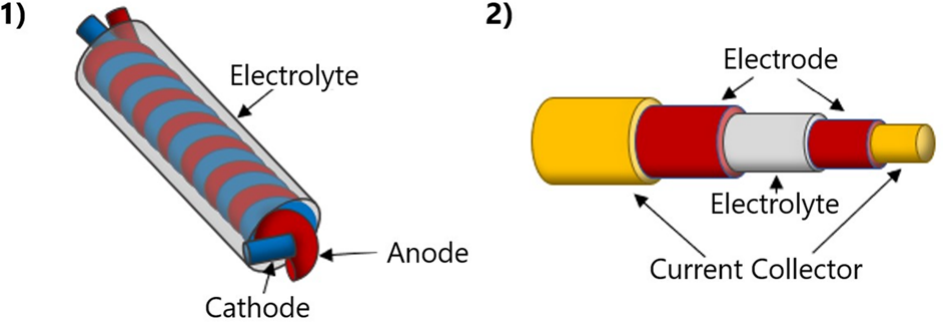
Representations of (1) twisted and (2) core sheath fabrication.

**Figure 6:**
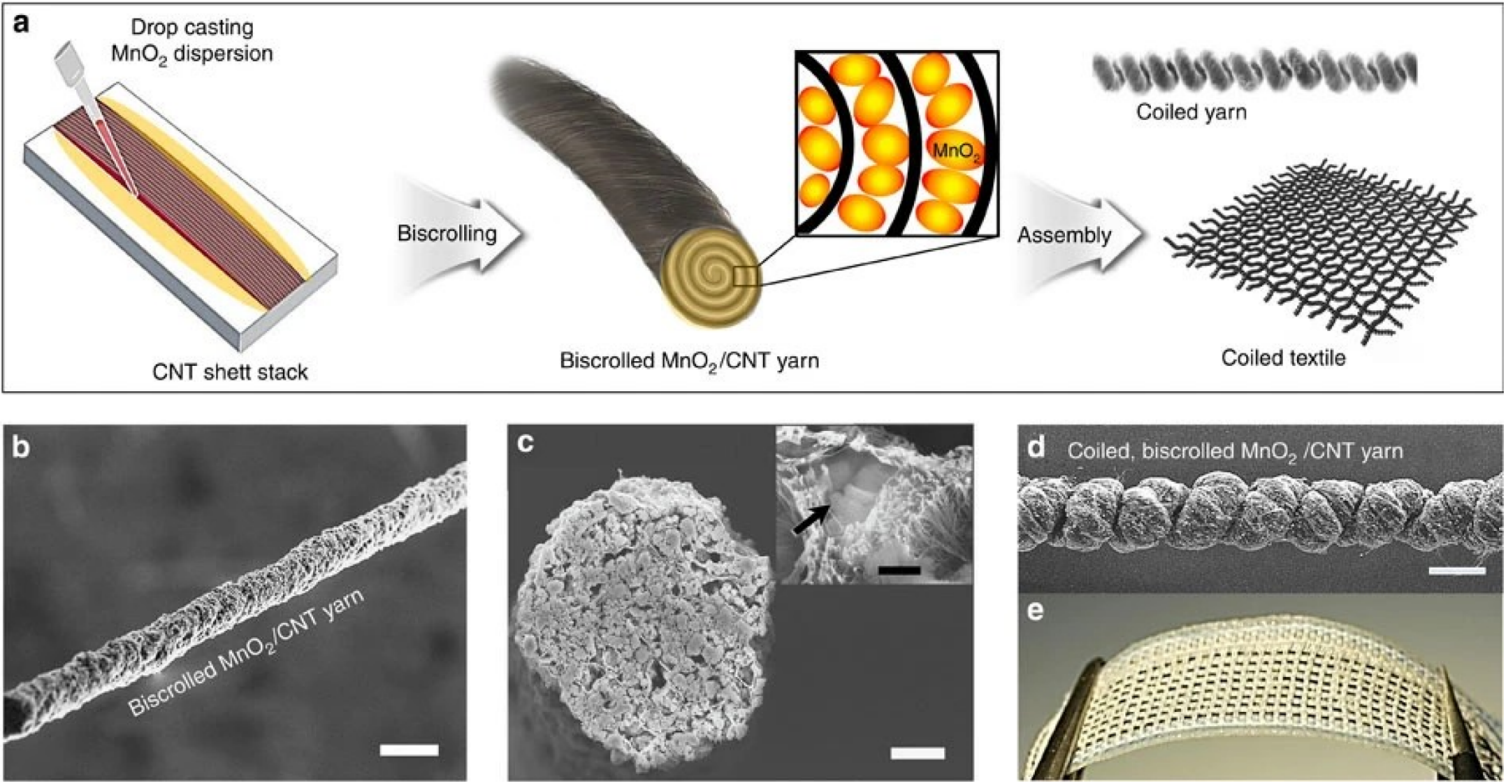
Biscrolled supercapacitor. (a) Fabrication; (b) SEM of yarn; (c) SEM of cross-section; and (d) SEM of Bi-scrolled yarn. Reprinted with permission from Choi, C.; Kim, K. M.; Kim, K. J.; Lepró, X.; Spinks, G. M.; Baughman, R. H.; Kim, S. J. Improvement of system capacitance via weavable superelastic biscrolled yarn supercapacitors. *Nat. Commun.***2016**, *7* (1), 13811. Copyright 2016, Nature Communications (CC by 4.0).

When coating a fiber/filament, researchers are able to create 3D supercapacitor structures from knitted or woven fabric. By utilizing conductive yarns/filaments, the device can exhibit intrinsic flexibility and stretchability.^64,65^ Singular yarns/filaments can become the cathode, anode, and electrolyte and can be knitted into textile supercapacitors, or they can become twisted and core sheath structures, as seen in Figure 5. Twisted is when the electrode filaments are wrapped around each other and then coated in the electrolyte so the electrolyte covers and separates them. This forms a singular filament which can be knitted, sewn, or embroidered onto a textile as it can exhibit excellent bending, tensile strength, and capacitive retention.^58,59,66−68^ Core sheath structures involve adding thin film layers onto the fiber/filament to create a layered supercapacitor. These systems offer high areal capacitance through the thin films, can be tailored to show faradaic responses, are flexible and lightweight, and have cycling stability.^69,70^ Zhao’s team in 2022 created a core sheath structure that featured extremely high mechanical flexibility and capacitance retention.^58^ Biscrolling is a fabrication method that utilizes both twisting and core sheath, as seen in Figure 6. The substrate is coated in the different conductive parts and then twisted in on itself to form a self-contained supercapacitor.^71,72^ This combined method can result in an asymmetric system with a high energy density of 43 μWh/cm^2^ and an areal capacitance of 322.4 mF/cm^2^.^72^

One of the drawbacks of using drop casting, bar coating, and dip coating includes making sure the fabric or yarn/filament is still able to deform and stretch for wearability. If the solution hardens too much, the textile will be brittle and unwearable. Therefore, the solutions can be partnered with polymers, such as PEDOT:PSS, to increase the coated textile’s flexibility.^61^ Another drawback is that the substrate may need to be coated multiple times to achieve the desired conductivity. This was shown by researchers Teixeria, Pereira, and Pereira when they had to coat their substrate up to nine times to get a stabilized supercapacitor.^63^ Finally, when creating a textile-based supercapacitor, a major concern is that the ink may degrade when worn or washed. Researchers have used electrolytes, polymers, binders, and resins to encapsulate or protect the ink so the conductive material does not deteriorate.^58,60^

Printing is an advancement in lean manufacturing because the amount of ink used in production is controllable. Printing also allows ink to be deposited in specific patterns onto the substrate. The two printing techniques utilized in textile supercapacitor fabrication are ink jet printing and screen printing. Ink jet printing is a popular, versatile type of printing that allows for the ink to be deposited in specific patterns with high accuracy via a cartridge much like a normal printer.^73^ This method is scalable, fast, and highly reproducible, allowing electrodes to be transferred to the substrate easily, and results in flexible, printed electrodes.^74^ Ink jet printing requires specific physical properties from the ink, such as low viscosity and small particle size, so that the cartridge is not clogged.^42^ Researchers have used ink jet printing to create supercapacitors on flexible substrates, with metallic or carbon-based inks doped with polymers being the most popular due to their flexibility, conductivity, stability, and electrochemical performance.^42,46,64,75−77^ The carbon materials offer high surface area which provides large electro-active sites for capacitance, and when doped with PPy prepared at subzero temperatures, researchers were able to demonstrate a high capacitance of 72.3 F/g while having good retention rates.^46^

Screen printing, another scalable and rapid printing technique, can be used to transfer the ink onto the substrate through a mesh screen, offering reduced material waste.^76,78^ Screen printing has a high deposition rate and, depending on the mesh construct and viscosity of the ink, offers varying printing quality onto the flexible substrate.^35,79−83^ Screen printing not only is functional but also can be aesthetically pleasing, as Zhang’s team showed by creating a butterfly pattern on a silk substrate that had a good capacitance of 19.23 mF/cm^2^ with a retention of 84% after 2000 cycles.^84^ Using polymeric material such as PANI allows the printed system the ability to deform and stretch without reducing the capacitance or mechanical strength.^85^ Another polymeric solution made from PPy/silver/manganese dioxide as the cathode and activated carbon as the anode was screen printed and stretched, twisted, crimped, and wound to show an excellent retention even after 40% strain.^47^

**Figure 7:**
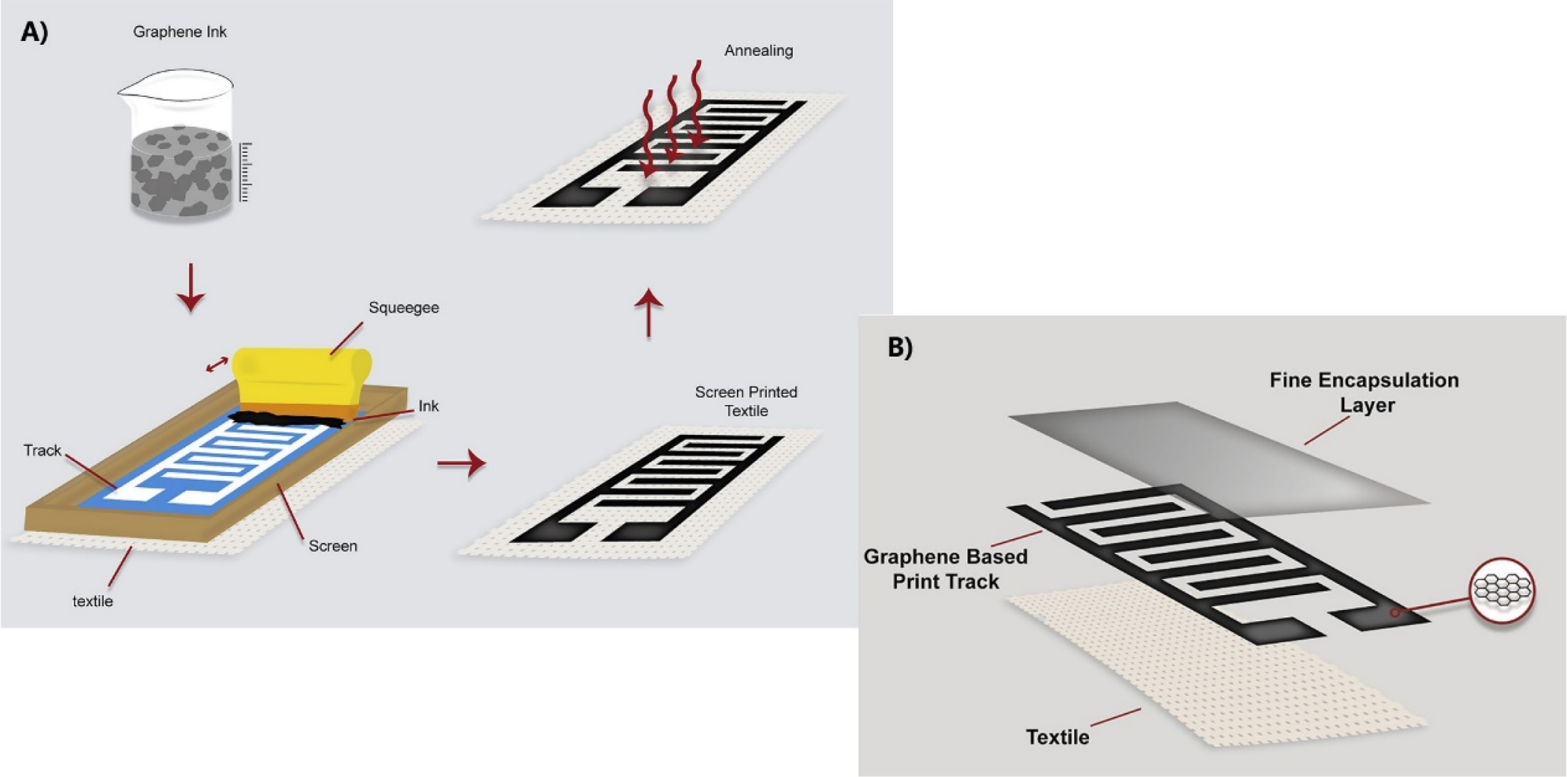
Screen printing with (a) schematic of the printing process and (b) encapsulation process. Reprinted with permission from Islam, M. R.; Afroj, S.; Beach, C.; Islam, M. H.; Parraman, C.; Abdelkader, A.; Casson, A. J.; Novoselov, K. S.; Karim, N. Fully printed and multifunctional graphene-based wearable e-textiles for personalized healthcare applications. *ISci.***2022**, *25* (3), 103945. Copyright 2022, IScience (CC by 4.0).

Concerns for printing include the ink drying out, creases or scratches from when the substrate contacts the printing device directly, homogeneity of the ink, and the poor resolution on screen printing that makes it unsuitable for small, precise designs.^86^ Therefore, careful determination of surface tension, flow of the ink, material dispersion, and specific rheological characteristics are required. However, printed textile supercapacitors have the opportunity to be highly conductive, washable, and flexible. Islam’s group demonstrated this by screen printing four layers of a pseudoplastic graphene ink and then encapsulating it so that it had the desired conductivity and washability for a textile supercapacitor, as seen in Figure 7.^87^

Vapor phase deposition is when particles are deposited in thin films onto the substrate under a controlled atmosphere which can result in a large areal capacitance.^88^ This method is simple, fast, and tunable and can utilize carbon, metal, or polymeric material. PEDOT is biocompatible and has been used with this method to create a supercapacitor with faradaic reactions.^89^ One research group even managed to maintain a capacitance retention of 101% after 10 000 cycles when PEDOT was deposited with graphene nanoplatelets.^48^ Spray coating also deposits thin films onto substrates that create rough surfaces, allowing for more surface area and increased potential for capacitance. Spray coating can also be flexible and stretchable when graphene or a polymer is deposited onto a stretchable fabric.^32,33,90,91^ Zheng et al. used both vapor phase deposition and spray coating to create a PEDOT/MXene supercapacitor which exhibited an extremely high specific capacitance of 1000.2 mF/cm due to the interconnected networks formed between the two materials on the fabric.^88^

In situ polymerization is widely used for preparing polymer-based nanocomposites with exfoliated structures. PPy is one polymer that benefits from in situ polymerization and can be added to a fabric substrate such as carbon fiber felt, pineapple polyester, water hyacinth polyester, and cotton to produce a flexible electrode that exhibits faradaic responses and almost no change in current after bending.^92−94^ Electrochemical polymerization is a method of depositing polymer films onto a substrate with the use of an electric field.^95^ This method can be partnered with other fabrication methods, such as dip coating, or it can be used on its own to create stretchable, flexible devices with high capacitance.^6,34,96^ Electrodeposition is where the deposition of material onto a conductive surface is controlled by electric current from a solution. Metals can be deposited onto conductive fibers, or conductive polymers can be added to metal substrates. One research group used carbon yarn and nickel–iron (NiFe) to weave a plain fabric that offered three electrodes, can degrade pollutants in water with a simple low voltage, and features a simple electrolyte deposition.^97^ Another group presented a low-cost supercapacitor formed from stainless steel mesh and electrodeposited PEDOT. This formed a 3D network that offered enhanced capacitance from its large surface area, and while this device did not match the cycling stability of carbon cloth supercapacitors, it did have a higher specific capacitance than other supercapacitors that used iron and reduced graphene oxide.^98^

Instead of coating or depositing the conductive material onto a substrate, the fiber/filament can be spun directly from the desired materials. This is done through several methods. First, researchers can use wet spinning to create their filament. Wet spinning is where the polymer dissolves along with other chemicals and then extrudes to form a filament which is then used in the textile system.^37,49,99,100^ These filaments can be highly stretchable and flexible and can be made from a combination of metal, carbon, and polymeric materials to offer both EDLC and faradaic responses.^51^ This process allows for researchers to control the properties the filament displays and can lead to highly efficient, conductive polymeric yarns. The spun fibers can be lightweight, flexible, and hollow, which gives good energy and power densities due to the large surface area and number of active sites.^101,102^ Wet spinning does not have any thermal degradation, and it can offer small fiber diameter; however, it requires solvents that can result in toxic remnants on the polymer if not properly rinsed. Therefore, careful treatment of the wet spun filament has to occur before the filament can be used in wearable textile devices.

Electrospinning is a spinning method that uses an electric force to draw threads of solution into a filament. This method allows tunable morphology, porosity, and composition while using very simple equipment.^50,103,104^ The ability to change the porosity of the filament means that filaments can be extruded with large pores to give higher electrochemical performance from the increased surface area, and the electrospun filaments do not need a binder to adhere the material to the substrate.^105^ This is valuable as store bought synthetic fabrics and filaments are often completely smooth and offer no pores to increase the surface area. One research group used this method to enhance the specific surface area of the device and achieved a specific capacitance of 396 F/g with a retention rate of 107% after 3000 cycles.^106^

**Figure 8:**
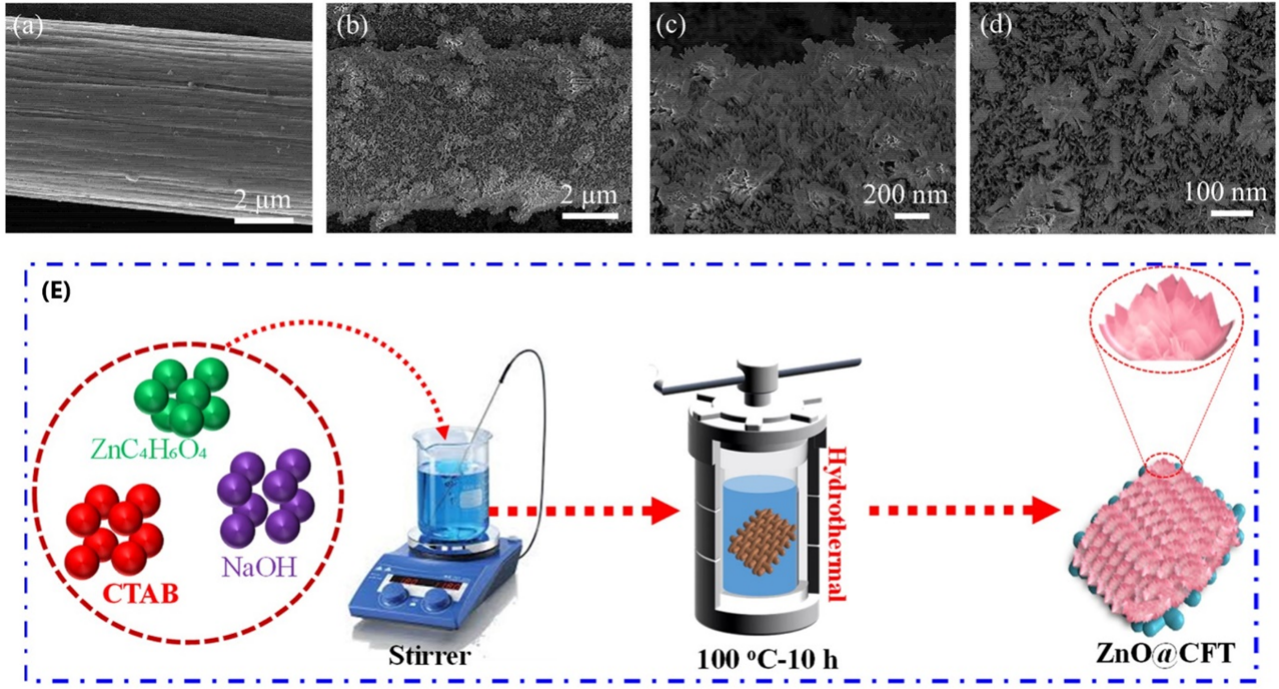
ZnO@CFT electrode. (a) SEM image of pristine CFT, (b) low-resolution FE-SEM image, (c) high-resolution FE-SEM image, (d) closer section from Figure 2c, and (e) schematic of the in situ process with ZnO nanoflowers. Reprinted with permission from Abbas, Q.; Javed, M. S.; Ahmad, A.; Siyal, S. H.; Asim, I.; Luque, R.; Albaqami, M. D.; Tighezza, A. M. ZnO Nano-Flowers Assembled on Carbon Fiber Textile for High-Performance Supercapacitor’s Electrode. *Coat.***2021**, *11* (11), 1337. Copyright 2021, Coatings (CC by 4.0).

Hydrothermal synthesis can be used to grow conductive material onto a substrate in order to create structures on the substrate that offer high surface area and faradaic responses.^107−111^ Hydrothermal synthesis can also be used along with a tube mold in order to form a self-assembled, fiber-shaped substance that can be used for high capacitance.^52,69,112,113^Figure 8 shows Abbas’s group using hydrothermal synthesis to form ZnO flowers on carbon fiber textiles that exhibit large surface area and active sites for electrochemical performance and a resulting high stability of over 90% after 3000 cycles.^109^ Solvothermal–coprecipitation synthesis is a method of forming a substance from chemical reactions in a solvent at high pressure and temperatures above boiling point. This method has been used to form a 3D net structure with a high specific capacitance of 1234.5 F/g and a retention of 83.7% after 1000 cycles.^31^

## 4 Materials

The material chosen to create the textile-based supercapacitor is critical, as the resulting device needs high conductivity, surface area, electron movement, and output power along with flexibility, stretchability, mechanical strength, and washability. Researchers have investigated metals, metal alloys, conductive polymers, carbon-based materials such as graphene, and other two-dimensional material such as MXene for the electrode.^19,23,114,115^ If the supercapacitor focuses on EDLC responses, carbon-based materials such as graphene, carbon nanotubes, and mesoporous carbon are desired because they are porous and offer large surface areas, which gives the supercapacitor more active sites to increase capacitance and cycling stability.^28^ Pseudocapacitor structures focus on conductive polymers and metal oxides or hydroxides, which offer high specific capacitance and faradaic reactions but low electrical conductivity.^31^ However, most textile supercapacitors combine both structures into a hybrid to utilize both types of materials and exhibit absorption and faradaic responses because they would not achieve the capacitance required to compete against other smart textile power systems.^116^ The chosen materials have potential for high electrical conductivity, energy and power densities, mechanical strength, flexibility, stretchability, and washability, properties that are fundamental to the performance of the system.

**Figure 9:**
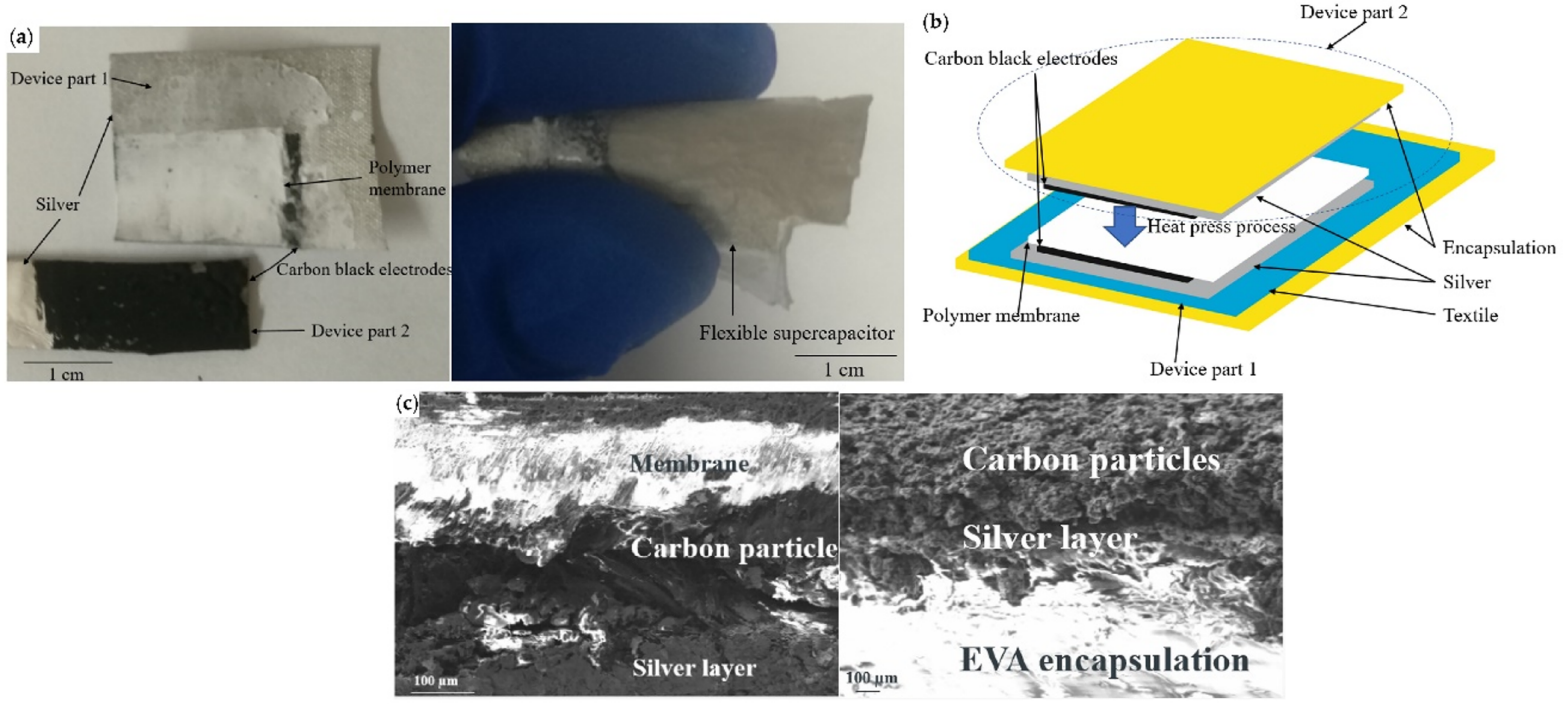
Supercapacitor with encapsulation. (a) Photo of split supercapacitor; (b) graphical representation; and (c) SEM images of electrode and encapsulation. Adapted with permission from Yong, S.; Beeby, S.; Yang, K. Flexible Supercapacitor Fabricated on a Polyester-Cotton Textile. *Proc.***2021**, *68* (1), 7. Copyright 2021, Proceedings (CC by 4.0).

Metals are ideal for supercapacitors as they are more conductive than polymeric materials and can lower or enhance the electron movement. Silver is a popular metal as it is the most conductive metal, and it can be used as both a wire, a nanoparticle, and in liquid form during fabrication.^77,81,117,118^ Even if silver is not used as part of the electrode, it can be used as the current collector, as shown in Figure 9,^81^ because it will maintain a low resistance between the electrodes and the sensor/actuator that is being powered. Silver can be used to boost the conductivity of other materials, such as graphene, to increase the conductivity but reduce the amount of metal in the electrodes.^118^ Copper is another conductive material that is used as the current collector because it has good stability, is durable, is soft, and offers good conductivity.^119^ While copper is usually preferred for lithium batteries because it will not intercalate with the lithium, it is also used in supercapacitors.^55,57^ Textile-based supercapacitors require the metals to be nontoxic, biocompatible, and not brittle; this includes MoO_3_, MnO, MnCO_3_, ZnS, ZnO, Ti_3_C_2_T_*x*_, Fe_2_O_3_, NiFe, and NiSe_2_.^57,63,90,107,109,111,120^ MoO_3_ exhibits excellent electrochemical capabilities, bending, and strain resistance when partnered with multiwalled carbon nanotubes to create a knitted device that can be removed for washing.^90^

While some metals can be used in their natural state, such as silver and copper, other metals require doping or the creation of compounds. Zinc is one metal that does not have a good electrochemical performance and therefore had to be altered in order to function as a supercapacitor. One research group doped zinc with carbon nanotubes to create a capacitance of 128.06 F/cm^3^.^58^ Other metals become oxides, and while metal oxides are not as conductive, they can be easily doped positive or negative to create the anode and cathode of the supercapacitor.^45,121−125^ One way of increasing the electrochemical responses and overall capacity of these metals is by creating ternary or quaternary compounds, substances made from three and four separate elements, respectively.^26,126^ A ternary compound made from GO/Ni/Cu exhibited an extremely high capacitance of 1220.5 F/g when paired with FeO/n-doped graphene.^123^

**Figure 10:**
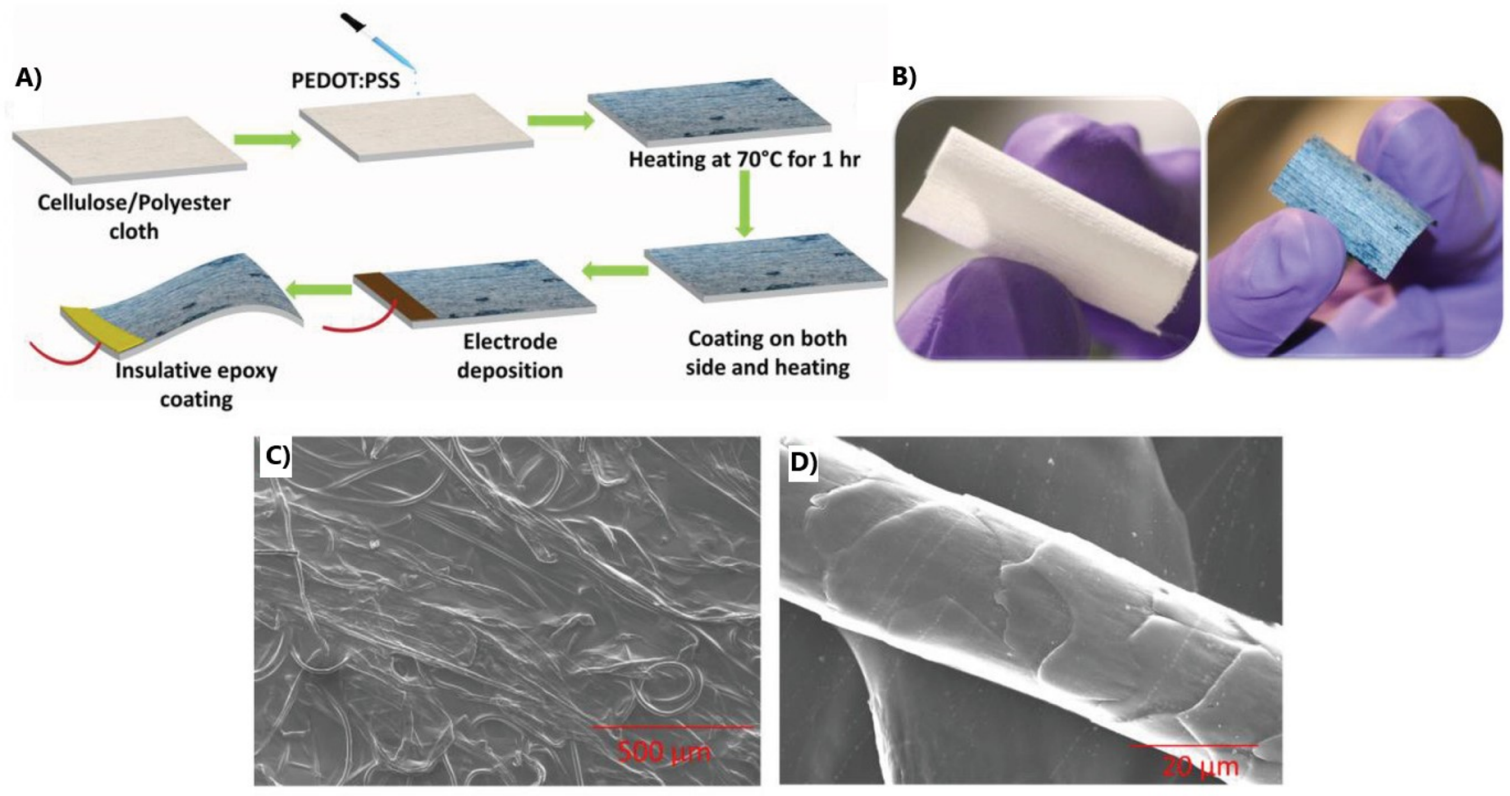
Coated cloth supercapacitor. (a) Fabrication method; (b) before and after photo of cloth; (c) SEM of coated cloth; and (d) closer SEM of cloth. Reprinted with permission from Manjakkal, L.; Pullanchiyodan, A.; Yogeswaran, N.; Hosseini, E. S.; Dahiya, R. A Wearable Supercapacitor Based on Conductive PEDOT:PSS-Coated Cloth and a Sweat Electrolyte. *Adv. Mater.***2020**, *32* (24), 1907254. Copyright 2020, Advanced Materials (CC by 4.0).

Metals can be brittle, which reduces their bendability and use as wearable devices, and tend to oxidize, which reduces their conductivity. Therefore, polymeric material is used as either a dopant or an alternative for both the electrodes and the current collection.^38,60^ While polymers do not offer high conductivity of metals, they have built-in structural flexibility, stretchability, and reduced brittleness that is helpful when making a wearable device.^61,102,108^ Conductive polymers also offer faradaic responses and can dope the metallic or carbon-based material to boost capacitance and energy densities.^21,127^ PANI, polypyrrole (PPy), and PEDOT:PSS are popular polymeric materials for supercapacitors because they are flexible, bendable, stretchable, conductive, and stable, offer faradaic responses, and have fast charge and discharge rates.^51,127−129^ PANI is affordable and easy to work with which makes it desirable for doping with other polymers, metals, or carbon material.^52,130^ PPy may have mechanical robustness and flexibility, but it has low cycling stability and is commonly used with other polymers or metals in order to overcome this disadvantage.^46,93,131−134^ The most popular polymeric material currently is PEDOT:PSS because it has high thermal and chemical stability and flexibility, is lightweight, offers a theoretical capacitance of 210 F/g, has an actual areal capacitance of 419 mF/cm^2^, and can be used as a method to bind carbon nanomaterial together.^21,48,52,100,135^ Manjakkal’s group designed a washable, sweat-based supercapacitor from PEDOT:PSS deposited on fabric, as seen in Figure 10, that offered an extremely low resistance of 7 to 22 Ω, good electrochemical performance, and stability after 4000 cycles.^44^

The most prominent materials being used in textile supercapacitors currently are carbon-based material including carbon nanofibers, carbon nanotubes, graphene, and graphene oxide. These materials have high specific surface area, which leads to better capacitance, high electrical conductivity, some flexibility, and good mechanical stiffness.^60,62,75,82,136^ However, carbon-based materials have limited capacitance and low energy density, and they are usually paired with a polymeric or transition metal to increase faradaic responses and overall performance.^32,51,60,61^ Carbon nanofibers possess more defects than carbon nanotubes, so they need to be added to another material to reduce the brittleness of the fiber.^137,138^ Carbon nanotubes can be either single or multiwalled depending on whether one or multiple graphite sheets are used to create the closed tube shape. Nanotubes are ideal for textile application because of their excellent mechanical and physical properties along with their chemical stability and electrical conductivity.^62,139,140^ Nanotubes have high surface area and porosity which elevates the number of active sites on the electrode and the overall electrochemical performance while being flexible and highly stable.^62,105,135^ Graphene and its derivatives offer some of the best contributions to textile-based supercapacitors because of their high conductivity and mechanical properties, modifiable surface chemistry, and large specific surface area.^60,72,100,113,141−144^ Graphene can be flexible, lightweight, and amalgamated with metals or polymeric material to create flexible, wearable supercapacitors.^45,145,146^ It can also reach extremely high capacitance and stability while being bent and stretched.^147,148^ Rao’s group used positively doped graphene as an electrode because of its porous morphology and flexibility to create a system that exhibited a high capacitance retention, stability, and long life.^149^

### 4.1 Substrates

Textile-based supercapacitors are fabricated from a conductive material being deposited on or forming a textile fabric, yarn, or filament. Using a fabric or yarn as the base for the supercapacitor has several advantages including intrinsic flexibility, deformation, tensile strength, and holding capabilities. Synthetic fibers may offer more stretchability, but they are smooth surfaces, which does not increase the surface area of the supercapacitor. Natural fibers, however, are porous and can attract and hold the ink to create large surface areas and improve ion storage capacity.^136^ Cotton is the most popular substrate currently because it is cost-effective, stretchable, flexible, hydrophilic, and porous, making it easy to buy, use, and create large surface areas or natural areas for ink permeation.^45,62,136,140,150^ Additionally, natural fibers such as cotton are able to stretch and bend when knitted or woven, and as long as the deposited material also has similar characteristics, the system will be wearable.^150^ Other materials that are utilized in textile supercapacitors include wool, which offers microporous structures for high specific surface area,^151^ cellulose and polyester,^44^ polyester mixes,^81,147^ polyacrylonitrile (PAN), and polypropylene (PP).^47,75^ If a polymeric material is extruded into a filament, the researchers can design the filament to be hollow or porous, which would create a larger surface area for active sites and give faradaic reactions. This tunable substrate can then be woven or knitted into a wearable fabric.^102,152^ Another substrate is carbon cloth, made from carbon fibers, which is low cost and offers high strength and flexibility.^101,152,153^ Textile supercapacitors are beginning to look at eco-friendly materials and ways to reduce the carbon footprint. Alzate’s group is working toward this by using pineapple polyester and water hyacinth polyester fabric as their substrate. Pineapple polyester is made from pineapple leaves and is popular in the Philippines, while water hyacinth is a highly invasive and harmful species; therefore, using this to form a textile can be beneficial to the ecosystem.^93^ These substrates are woven into the textile and, while currently bolstered with polyester for higher tensile strength and showing low stability in its capacitance cycling, show that there are many new substrates that are acceptable for sustainable, textile supercapacitors.

### 4.2 Electrolytes

Electrolytes function as the area between the two electrodes that transfers and balances the charge between the two electrodes and can be either liquid or solid-state.^154^ While liquid electrolytes offer excellent conductivity, they have several weaknesses including potential shunt currents, fire hazards, low ionic preciseness, and the inability to be used for textile supercapacitors.^155^ Solid-state electrolytes, on the other hand, can be solid or gel, and the gel behaves as a solid due to the electrostatic forces holding the polymer structure together.^156^ These solid-state electrolytes can be thin and flexible, but gel electrolytes suffer from low ionic conductivity because of their viscous nature.^141,154^ For use in textile application, a gel or solid-state electrolyte is required. To help increase the capacitance of the system, a thin-film electrolyte should be used to maximize the surface area that contacts the electrode.^89^

Electrolytes are composed of a polymer material due to its flexibility. This can include poly(ethylene oxide) (PEO),^157^ polyacrylonitrile (PAN),^158^ poly(methyl methacrylate) (PMMA),^158^ polyacrylamide (PAM),^159^ poly(ethylenimine) (PEI), polyacrylic acid (PAA),^160^ poly(vinylidene fluoride) (PVDF),^37^ and poly(vinyl alcohol) (PVA). PVA is stable, transparent, and nontoxic and has good ionic conductivity, characteristics necessary for textile application.^161,162^ However, because polymeric materials feature low ionic conductivity, researchers pair them with an ionic compound to boost their ionic conductivity and boost the efficiency of the system.^163^ Ionic compounds that have been added to these polymeric materials include sulfuric acid (H_2_SO_4_),^51,65,69,88,106^ phosphorous acid (H_3_PO_3_),^54^ potassium hydroxide (KOH),^113^ potassium chloride (KCl), potassium ferricyanide (K_3_[Fe(CN)_6_]),^29,164^ 1-ethyl-3-methylimidazolium tetrafluoroborate (EMIMBF_4_),^37^ sodium chloride (NaCl),^165^ and bacterial cellulose.^159^ However, one of the main concerns with electrolytes is that some may be poisonous or dangerous to humans when incorporated into wearable devices, so biocompatibility is required.^55^ One way is to use material that is an electrolyte but digestible, such as isotonic or energy drinks that have been made into a gel.^166^ These gels are consumable and biocompatible but do not have the potential for high power application at this time. Another way is to use a keratin-based solution; however, because the keratin has poor fiber forming ability, it had to be electrospun and cross-linked with PET.^167^ A more successful way to negate the potential for toxicity is by using human sweat as the electrolyte.^44^ A group led by Selvam created a textile supercapacitor that used sweat as the electrolyte and was able to achieve an outstandingly high capacitance retention of 92% after 50 000 cycles. The system was bonded with tape to the human skin so that the sweat could be utilized and also showed high tensile strength and the ability to be worn while exercising.^168^

**Figure 11:**
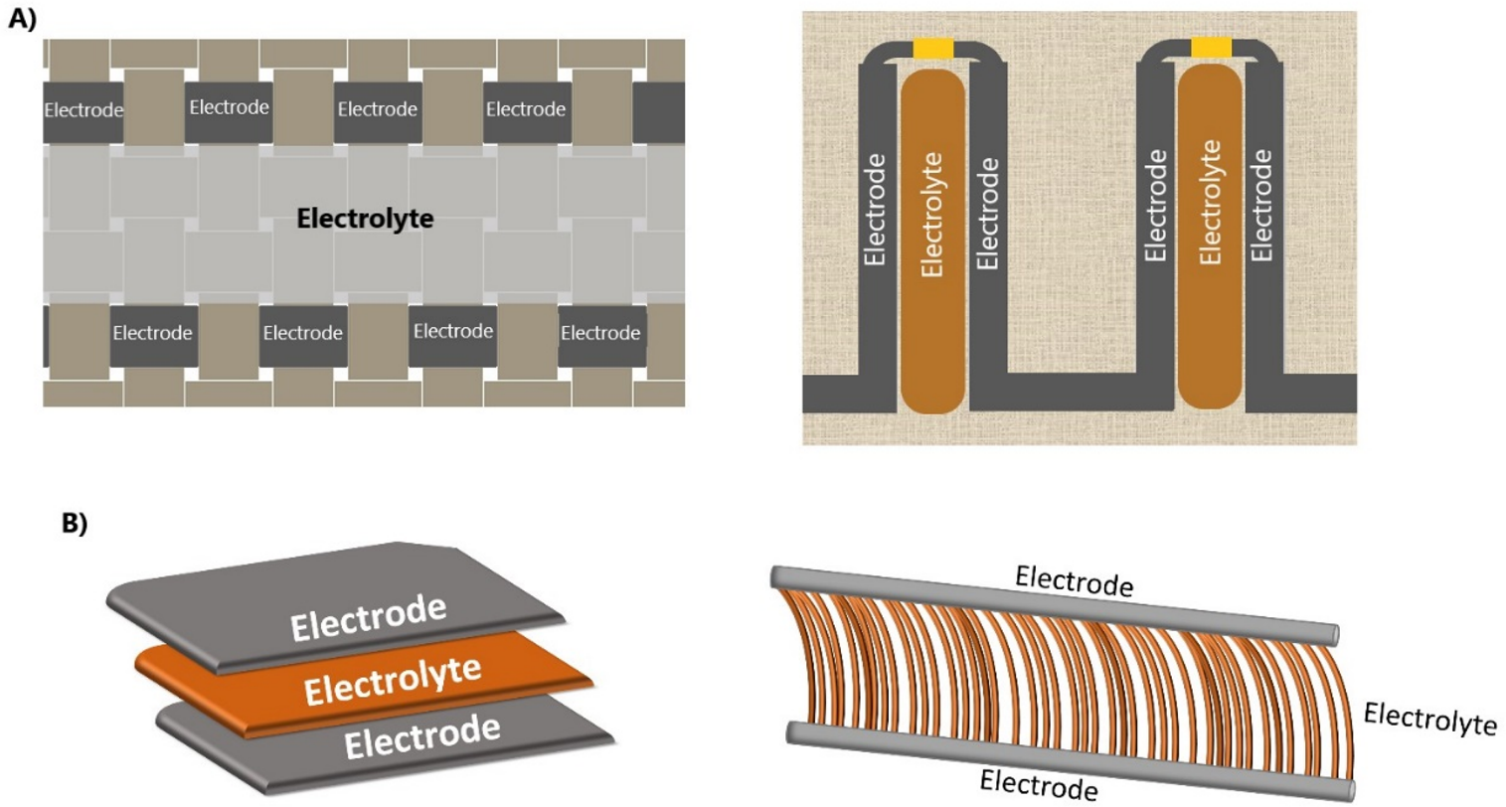
Representations of (a) 2D and (b) 3D integration of supercapacitors into a textile substrate.

Various 2D and 3D methods can be used to fabricate the electrolyte onto a substrate. For 2D, the electrodes and electrolyte are distributed horizontally next to each other. For 3D, the system is vertical, as seen in Figure 11. An example of 2D designs is coating or casting a thin gel electrolyte onto the textile substrate between the two electrodes.^67,169^ This application is one of the easier methods because the electrolyte can be added before or after the electrode assembly. A potential drawback for this method is that the electrolyte may not extend the full space between the electrodes. An example is if the electrodes are carried through the textile and not just found on the surface, the electrolyte will not be situated fully between them and will interrupt the system’s electron flow. Therefore, using a porous textile or yarn that allows the electrolyte to be incorporated deeply within the fibers and allows more usable surface area for the electrodes can lead to an increase in capacitance.^67^

For 3D integration, the electrolyte becomes sandwiched between the two electrodes on a vertical plane. This can become bulky if not fabricated correctly and has been successfully proven in twisted and core sheath methods.^65,70,71,89^ While the biscrolled method has also been used, the electrolyte becomes external to the electrodes but allows the electrolyte and electrically conductive material to maintain the necessary close contact.^71^ A third way of creating a 3D supercapacitor is by using a spacer knit fabric. This fabric has two surfaces that are separated by a filler yarn and offers excellent resilience to compression and structural integrity. In this structure, the electrodes can be fabricated onto the outer knit structures, and the separating yarn is the electrolyte. One research group created an all-textile supercapacitor from this method by using three types of coated yarn to form an asymmetric system: one yarn was coated as an anode, one as a cathode, and the final one as the electrolyte.^170^ Another group braided the electrode material to form a dense, protective layer around the electrode.^171^

## 5 Current Challenges and Future Applications

Textile supercapacitors offer new avenues of research for smart textiles. However, there are still some limitations that need to be addressed before commercialization of these devices can occur. First, most of the research done on textile supercapacitors does not focus on washability. Textile supercapacitors that are wearable will need to be able to withstand standard washing procedures before they can be applied in any industry successfully. Researchers have noted that the poor stability of the deposited inks on textile substrates with regard to washability is a primary challenge for commercialization of these systems, but many researchers do not answer how to prevent this degradation from happening.^45,75,87,168^ While one research group decided removing the device from the textile before washing to avoid the degradation was necessary,^90^ most groups decided to encapsulate the system. Researchers used post treatments of PDMS, PU, resin, or other polymeric material to encapsulate the system.^62,68,81,83,96,143^ Even after encapsulating, however, rarely did the research group report their findings on how well the encapsulation held up from washing. One group used silicon rubber encapsulation to protect the system and saw no decrease in electrical output and was able to continue bending the device,^70^ while another group used a resin and saw a slight increase in resistance after washing.^44^ A third group used a thin, stretchable PU-based encapsulant over their device. This was proven effective as, after the first washing, the resistance was only 1.5 times higher than the initial number and after 10 washings only 3.5 times higher.^87^ This was compared to the system without encapsulation which resulted in double resistance after one wash. While encapsulation is a viable option to protect the system from degrading, it is necessary to choose techniques that will not negatively affect the tensile properties of the device, such as deformation, stretch, and even comfort.

A smaller challenge is how to match batteries or nontextile supercapacitors in performance. Currently, textile supercapacitors are not able to match textile batteries, and one reason may be because the way the device is designed to move the electrical current needs to be improved. The capacitance potential of the system may be lost due to placement of the current collectors. The current collects and conducts the electrical current from the electrodes to the power source and the sensors. Usually these collectors are placed on the ends of the electrodes, but as one research group pointed out, the longer electrodes may store more charges; however, if the collectors are on the ends of the electrodes, then resistance and current collection can be negatively impacted.^94^ They found that placing the current collectors in the middle of the electrode or having multiple current collectors for one electrode reduce resistance and increase capacitance. This optimization of the system may help current textile supercapacitors improve their capacitance and elevate their potential for being commercialized.

A third challenge is to make the textile supercapacitor truly biocompatible. Most of the materials used in fabricating these devices are said to be nontoxic to humans, but very few research groups have actually tested the biocompatibility of the system. Selvam and Yim did a biocompatibility test on their supercapacitor by examining all of their material against HT-29 cells.^111^ It is not enough to claim that the materials and fabrication methods are nontoxic, and tests have to be done to verify these claims. One research group pointed out that some electrolytes may not be safe for humans, but biocompatibility tests on the electrolytes are rarely seen in this research.^55^ Since these systems will be touching human skin, it should be a requirement that compatibility tests are run before commercialization.

A final challenge is that current fabrication methods are varied and either expensive or difficult to scale up for manufacturing. While some materials, such as cotton, can be affordable, conductive materials such as PEDOT:PSS or graphene can be expensive. The final cost and access to equipment to make the devices will determine if companies are willing to manufacture the textile supercapacitors. Textile companies may be hesitant to purchase new machines to meet specific fabrication methods, but they may be more willing to manufacture the system if they already have the machine and only have to order one part or specific material. Because different countries and companies have different machines, one fabrication method may not work, so knowing what manufacturing process will be most successful and where will be important to seeing textile supercapacitors be commercialized.

Once these challenges are addressed, textile supercapacitors have the opportunity to change the future of smart textiles. These changes are for not only future applications of these systems but also how smart textiles are fabricated. The research shown in this manuscript shows that unique substrates can be used to make supercapacitors. Textile supercapacitors do not have to be made from a singular substrate, such as cotton. These supercapacitors can be fabricated on local flora, such as pineapple leaves and bamboo, invasive flora, such as water hyacinth, local fauna, such as wool, and even man-made fibers such as nylon or even a designed filament from conductive polymer. This allows different countries to find which manufacturing process will be most effective for them.

Textile supercapacitors have the ability to change industries. As an energy storage system, they can be fabricated onto medical garments, sport clothes, and functional clothes.^60^ Medical garments that are currently only provided in hospitals or doctor’s offices can become at-home medical treatments so that the patient is not confined to a medical space. Already supercapacitors can wirelessly transfer data every 30 s for 96 min without needing a charge,^57^ so doing some at-home care or analysis is possible. Sportswear can monitor physiological responses when at the gym or in a match, allowing the person exercising or the medical officer information about the performance of the athlete. By adding elements such as Mn to a textile supercapacitor, the textiles can become powered reflective safety wear from the fluorescence found in the element.^63^ Additionally, this device can be partnered with an energy capture system to become a self-sufficient smart textile. Researchers have already paired textile supercapacitors with triboelectric systems to control heat and monitor the human body,^58^ but they can go further. Self-reliant smart textiles can give communities that do not have access to conventional electrical means a way to power simple electronics and lights through wearing a garment that can capture and store the electrical energy.

## 6 Conclusion

This review shows that textile supercapacitors have advanced in the recent years, and new fabrication methods and materials have enhanced their stability, energy and power densities, deformation, and stretchability. While there are still some challenges with washability, manufacturing, and getting the systems to perform at the same level as a battery, these devices have strong life cycles and are wearable. Textile supercapacitors can be fabricated onto a textile or a yarn or even manufactured into a designer filament in order to meet the standards required for excellent capacitance. By using a hybrid design and combining EDLC and pseudocapacitor material, they feature increased surface area from porous materials, highly conductive compounds, and polymers with faradaic responses. Combining metal, polymers, and carbon-based material, high capacitance is exhibited while maintaining the tensile characteristics that make the textile wearable. Deformation and stretchability may be inherent in textile substrates, but not all inks lend themselves toward those qualities; therefore, depositing the ink correctly and with the right material is critical to creating a textile supercapacitor that is viable for commercialization. These devices have numerous applications in industries from medical to sport and are the future of self-sufficient, smart textiles.
